# Quality of life in elderly people at the start of using in-home care

**DOI:** 10.1186/s40064-015-1161-x

**Published:** 2015-07-28

**Authors:** Miyuki Imanishi, Hisao Tomohisa, Kazuo Higaki

**Affiliations:** Research Institute of Rehabilitation Science, Osaka Prefecture University, Habikino 3-7-30, Habikino-shi, Osaka Japan; Department of Psychiatry, Kyoto University Hospital, Shogoin-Kawaramachi 54, Sakyo-ku, Kyoto Japan; Department of Community Health, Osaka Prefecture University, Habikino 3-7-30, Habikino-shi, Osaka Japan

**Keywords:** In-home care services, Elderly people, QOL

## Abstract

**Background:**

Quality of life (QOL) among 200 elderly people planning to use in-home care services available to those with severe illness was surveyed to identify the most effective method of improving QOL of the elderly through such care services.

**Results:**

QOL was surveyed using a PGC Morale Scale, and factors related to QOL were verified by multivariate analysis. The most relevant factors for QOL among the 11 analyzed were the client’s reason for choosing to use in-home care services and the client’s family structure. The average PGC-MS score was 9.09 ± 2.6 (mean ± standard deviation). Analysis of factors significantly affecting PGC-MS score identified treatment history and job type (p < 0.001), family structure (p < 0.009), age (p < 0.008), and years of education (p < 0.029). In addition, after performing multiple regression analysis using treatment history, years of education, employment history, and family structure as independent variables, treatment history (visiting a hospital and receiving treatment but deciding to use in-home care services later) remained significantly related to PGC-MS score (p < 0.001), as did family structure (three-generation families; p < 0.001). Further, with regard to treatment history, PGC-MC scores were higher in those who visited a hospital and received treatment but decided to use in-home care services later than in those who decided to enroll in the care service while still in the hospital. In contrast, with regard to family structure, PGC-MS scores decreased in the order of three-generation families, those living alone, couples, and two-generation families.

**Conclusions:**

QOL of elderly people might be improved by the provision of in-home care services with consideration of consumers’ intentions and factors known to influence QOL.

## Background

The 2012 version of the Annual Report on the Aging Society released by the Cabinet Office placed the total population of Japan at 127.8 million in 2011, with the elderly population (aged 65 or older) totaling 29.75 million, the highest number ever recorded (Cabinet Office, Government of Japan 2012). The ratio of people aged 65 or older to the total population, known as the population aging rate, was thus calculated as 23.3% for Japan. Given that, as of 2013, this value is the highest globally, Japan may be termed a “super-aging society” (Cabinet Office, Government of Japan 2012). The Cabinet Office’s estimates suggest that the elderly population in Japan will continue to grow, with the aging rate reaching 39.9% in 2060—meaning that two out of every five people will be aged 65 years or older (Cabinet Office, Government of Japan 2012). To address problems facing aging societies, the Japanese government has adopted measures to reduce medical expenses among the elderly, including the establishment of a long-term care insurance system and the revision of medical fees. Elderly care in Japan is also currently shifting from hospital care to community-based and in-home care. However, several issues have been reported concerning the quality of in-home care in Japan.

Kawai points out that the social norms of a super-aging society tend to regard the elderly as a social problem that needs to be addressed (Kawai 2011), and the number of people who can live happily knowing that they are considered a social problem is questionable. Given Washida’s assertion that humans cannot thrive without recognizing some sort of meaning in their lives (Washida 2003), we must strive to understand the role of nursing care in preventing elderly people from losing meaning in their lives in a super-aging society and the kind of society in which elderly people can live with a sense of happiness. Seeking such understanding may help improve the welfare of people of all generations.

In Japan, under the Nursing Care Insurance Law, individuals aged 65 or older who have been diagnosed by a physician with an illness inflicting undue burden on daily living and meriting assistance from an outside source may apply for use of an in-home care service, subsidized by public funds, to reduce the care burden on their family or supplement their own personal care. Japan has three types of in-home care services. One type is a service in which physical therapists and occupational therapists visit the homes of seniors and provide rehabilitation services. This service is usually provided once or twice a week, and one therapy session lasts for 60 min. The second type is a service in which nurses provide services such as medication management, blood sugar level measurement, infusion services, and wound disinfection at seniors’ private homes. This service is also provided once a week or, depending on the situation, a few times a week by nurses under instructions given by a doctor. The third type is a service in which caregivers visit seniors’ private homes to perform housekeeping services and provide personal assistance. Depending on the severity of client’s symptoms, a variety of services are provided; in some cases, only housekeeping services—such as shopping, cleaning, washing clothes, and cooking—are provided, while in other cases, the caregivers will bathe the clients, change their diapers, and assist them with changing clothes and eating meals. Visiting frequency for this third type of service also depends on symptom severity, ranging from once a week for those with mild symptoms to daily visits for those with severe symptoms. Seniors and their families can choose the hours and number of days per month for receiving these three types of in-home care services within the usage limit of the subsidy system established by the national government. These in-home services provide temporary nursing at home for dysfunctional elderly individuals, including visitation, evaluation, and rehabilitation. However, tailoring in-home care services to each individual to ensure the best care and improve quality of life (QOL) can be a daunting task.

Here, to provide a basis on which ideal in-home care services can be designed in order to improve the quality of life (QOL) of elderly people, we studied the QOL of individuals who were planning to use in-home care services and analyzed factors influencing QOL of the elderly.

## Research method

### Research Design

A cross-sectional analysis was conducted to evaluate QOL of 200 elderly people (age ≥ 65 years) who were planning to start using in-home care services.

### Research participants

Potential participants were 223 individuals who had recently signed a contract to receive service from any of 18 in-home care service locations in the Kansai area of Japan between April 1 and June 30, 2013. After exclusion based on criteria described below, the study population consisted of 200 elderly people aged 65–84.

### Selection/exclusion criteria

Our study focused on elderly people seeking to use in-home care service due to severe illness, as this is the major criterion for being allowed to use these public-subsidized in-home care services. Individuals who were either presently hospitalized and about to be discharged or who had recently (within the past 3 months) been hospitalized for an illness covered by the Nursing Care Insurance Law and who had a general status stable enough to allow participation in the survey were selected. Those with a score on the Mini-Mental State Examination (MMSE) of 24 or lower were excluded.

### Data collection

The following background and clinical data of participants were collected from either medical records or the home care service contract: age, sex, disease category, treatment history, degree of required care, family structure, job type (including homemaker and part-time employment), years of education, hobbies, and religious activities.

QOL of participants was assessed using the Philadelphia Geriatric Center Morale Scale (PGC-MS). The PGC-MS was developed by M. P. Lawton in 1972 as a scale to measure happiness in the elderly and is used in studies worldwide to evaluate QOL among elderly people. Higher scores on this scale have been shown to indicate higher QOL. Our present survey used the validated Japanese version of the PGC-MS, developed by Daisaku Maeda. Score is assessed via questionnaire with 14 “yes/no” questions three questions with alternately-phrased binary answers, with answers in the “yes” column given 1 point and those in the “no” column given 0 points, for a maximum of 17 points. Higher scores indicate higher quality of life. The suitability of the PGC-MS for elderly people living in care facilities or at home has been confirmed (Koyano 1996). The PGC-MS score was used as the main outcome of this study. The questionnaire was completed by the participant alone or with help from a family member or friend.

Participants’ degrees of mental and physical independence were assessed by specialists (such as occupational therapists) using the functional independence measure (FIM), which is a measure commonly used to assess current activity of daily living (ADL). FIM evaluates motor and cognitive functions based on responses to 18 items over 7 steps (range of total possible scores: 18–126) and is mainly used at rehabilitation clinic sites, both in Japan and abroad. Higher scores on this scale have been shown to indicate higher ADL function.

### Analysis

Under double-blinded conditions, a researcher who was different from and independent of the authors analyzed the survey data using SPSS Ver. 22.0. Analysis used 11 variables (age, sex, disease category, treatment history, degree of required care, family structure, job type, years of education, hobbies, religion, and FIM score) as independent variables, and relevance to PGC-MS was verified using multiple regression analysis. The PGC-MS score was then used as an assay variable. Among the independent variables, the available disease categories were musculoskeletal system disease, nervous system disease, cardiopulmonary disease, trauma, systemic disease, or immune disease. The “treatment history” was regarded as the subjects’ experiences with therapy which led them to use an in-home care service and was categorized as people who planned to use in-home care services immediately upon leaving the hospital and those who had already visited a hospital and received therapy but had come to require the use of in-home care services. “Job type” was categorized into clerical work, sales, specialist job/engineer, service, agriculture/forestry/fishery, production/labor services, transportation/communication, and other. “Family structure” was categorized into single/living alone, married couple, two generations living together, and three generations living together. In the analysis of variables influencing PGC-MS, the Mann–Whitney U test was used for sex, treatment history, hobbies, and religion, while the Kruskal–Wallis test was used for disease category, career, and family structure. The significance of age and years of education were evaluated based on Pearson’s product-moment correlation coefficient, and that of the degree of required care and FIM based on Spearman’s rank-correlation coefficient. In multiple regression analysis, stepwise selection was conducted (standard: probability of F to be imposed ≤0.050; probability of F to be excluded ≥0.100). Analysis was then conducted by including only those variables deemed significant in individual univariate analysis. The Shapiro–Wilk test was used for validation of regular date distribution. Significance was set at a level of 5%.

### Consent and ethical approval

The purpose of our research was explained orally and in written form to participants, and their informed consent was obtained. We received ethical approval to conduct this study from the Research Ethics Committee of Osaka Prefecture University (Approval no. 2012-OT17).

## Results

### Participant characteristics

The average PGC-MS score among all participants was 9.09 ± 2.6 [mean ± standard deviation (SD)], a value less than the findings of Maeda in healthy elderly men in Japan (mean ± SD 11.97 ± 3.57) (Maeda and Asano 1979). Our research population of 200 participants included 79 men and 121 women, with a mean ± SD age of 78.13 ± 5.1 years. Factors influencing the PGC-MS metric scale are described in full in Table 1. Mean ± SD for years of education was 10.95 ± 2.3, required level of care was 2.65 ± 1.2, and FIM score was 84.73 ± 20.4. With regard to factors influencing the PGC-MS nominal scale, 48% of survey respondents intended to start using care services immediately after discharge, with 52% starting later. A slim majority reported engaging in hobbies (55.5 vs. 44.5%), and most considered themselves religious (59.5 vs. 44.5%). Most respondents lived either as a couple with a spouse (38.5%) or alone (29.5%), with smaller proportions living in two- (16%) or three-generation (16%) homes. Regarding disease category, most were afflicted with diseases of the nervous system (32%), followed external wound such as fractures (16.5%), and diseases of the musculoskeletal system (15.5%). Most respondents had worked in the forestry and fishery industries (24%), followed by manufacturing and labor industries (17.5%) and technical professions (17%).

**Table 1:** Analysis of factors influencing PGC-MS: metric scale

Variable	Mean value	Standard deviation	Correlation function (a)	Significance probability (*P/*b)
Age	78.13	5.175	0.188	0.008**
Degree of required care	2.65	1.207	−0.052	0.464
Years of education	10.95	2.392	−0.154	0.029*
FIM	84.73	20.412	0.124	0.080

Mean value and standard deviation of the degree for required care and FIM indicate median and inter-quartile range.

*FIM* functional independence measure.

Spearman’s ρ was used for correlation function (a), and the two-tailed test was used for significance probability (b).

### Correlation coefficients

Correlation coefficients were as follows: sex (U = 4195.0, Z = −1.473), treatment history (U = 2482.0, Z = −6.188), hobbies (U = 4244.0, Z = −1.724), religious activities (U = 4312.5, Z = 1.272), disease category (X2 = 6.226, F = 5), job type (X2 = 28.189, F = 7), family structure (X2 = 11.85, F = 3).

### Variables Influencing PGC-MS

Treatment history and job type were both significantly associated with PGC-MS score (p < 0.001), as was family structure (p < 0.009). However, no significant association was noted between PGC-MS score and sex, disease category, presence of hobbies, or engagement in religious activities. PGC-MS scores for job type increased in the order of agriculture/forestry/fishery, transportation/communication, service, clerical work, specialist job/engineer, and production/labor services. Multiple regression analysis to evaluate the relevance of the 11 background factors with PGC-MS showed significant relationships between QOL and the following 5 factors: “treatment history” (β = 0.438, p < 0.001), “job type” (β = −0.02, p < 0.001), “family structure” (β = 0.274, p < 0.009), “age” (β = 0.065, p < 0.008), and “years of education” (β = −0.053, p < 0.029) (Tables 1, 2). On additional multiple regression analysis using these five factors as independent variables to evaluate their relevance with PGC-MS, only two retained a significant relationship with QOL: visiting a hospital and receiving therapy but deciding to use in-home care services later (p < 0.001), and having a family structure including three generations (p < 0.001) (Table 3). Further, with regard to treatment history, PGC-MC scores were higher in those who visited a hospital and received treatment but decided to use in-home care services later than in those who decided to enroll in the care service while still in the hospital. In contrast, with regard to family structure, PGC-MS scores decreased in the order of three-generation families, those living alone, couples, and two-generation families.

**Table 2:** Analysis of factors influencing PGC-MS: Nominal scale

Factor	Comparison group	Frequency	Percentage	Median^a^	Inter- quartile range^a^	Mean rank	Significance probability (*P*)
Sex (a)	Female	121	60.5	9.0	4.0	105.3	0.141
	Male	79	39.5	8.0	3.0	93.1	
Disease category (b)	Musculoskeletal system	31	15.5	10.0	4.0	105.85	0.282
	Nerve system	64	32.0	8.0	3.0	93.70	
	Cardiopulmonary disease	25	12.5	8.0	2.0	82.18	
	Trauma	33	16.5	9.0	3.5	106.76	
	Systemic disease	25	12.5	10.0	4.0	106.82	
	Immune disease	22	11.0	10.5	5.3	117.00	
Treatment history (a)	Hospital discharge → In-home care	96	48.0	7.0	3.0	74.4	0.001***
	Hospital visit → In-home care	104	52.0	10.0	3.8	124.6	
Job type (b)	Clerical work	14	7.0	8	4.3	88.18	0.001***
	Sales	19	9.5	10	5.0	125.92	
	Specialist job/engineer	34	17.0	8	3.3	87.79	
	Service	4	2.0	9	4	102.00	
	Agriculture/forestry/fishery	48	24.0	10.5	4	124.57	
	Production process/labor services	35	17.5	7.0	3.0	64.93	
	Transportation/communication	9	4.5	9.0	3.0	104.56	
	Other	37	18.5	9.0	2.5	105.05	
Hobby (a)	Have hobbies	111	55.5	8.0	3.0	94.2	0.085
	No hobbies	89	44.5	10.0	5.0	108.3	
Religious (a)	Yes	119	59.5	8.0	3.0	96.2	0.204
	No	81	40.5	10.0	5.0	106.8	
Family structure (b)	Single/living alone	59	29.5	9	5	107.23	0.009**
	Married couple	77	38.5	8	3	92.86	
	Two generations	32	16.0	8	3.8	81.36	
	Three generations	32	16.0	10.0	5.0	125.61	

*** *P* < 0.001; ** *P* < 0.01.

Mann–Whitney U test was used for factors (a) and Kruskal–Wallis test for factors (b).

^a^Median and interquartile ranges refer to PGC-MS scores.

**Table 3:** Stepwise multiple regression analysis of factors influencing PGC-MS

Factor	Category	Partial regression coefficient	Standard partial regression coefficient (β)	Significance probability (*P*)	R^2^	Adjusted R^2^	VIF
Treatment history	Hospital visits → in-home care (a)	2.247	0.438	0.001***	0.269***	0.256***	1.000
Family structure	Three generations (b)	1.635	0.274	0.001**			1.000

*** *P* < 0.001; ** *P* < 0.01.

The standard for category (a) was Hospital discharge → In-home care, and that for category (b) was single-living.

## Discussion

Here, we evaluated the QOL of elderly people planning to start using in-home care services and assessed relevant factors influencing QOL. The average PGC-MS score among all participants was 9.09 ± 2.6 (mean ± standard deviation), a value below that reported by Maeda in a previous study in healthy elderly people (Maeda and Asano 1979) and which supports our hypothesis that presence of disease and impairment of physical function negatively influences QOL among the elderly. We also evaluated the relevance of the 11 factors of age, sex, disease category, treatment history, degree of required care, family structure, job type, years of education, hobbies, religion, and FIM score with PGC-MS. Findings from univariate and multivariable analyses showed that, of these 11 factors, treatment history and family structure were the only two significantly influencing PGC-MS score.

In the present study, the variable of “treatment history” was divided into two categories of people: those who were planning to use in-home care services immediately after leaving the hospital (48% of total population), and those who had already visited the hospital and received therapy but had later come to require the use of in-home care services (52% of total population). While these proportions were nearly equivalent, this difference in history of how subjects came to use in-home care services greatly influenced their QOL.

Mean QOL score among subjects who began to use in-home care services immediately after hospital discharge was significantly lower than the mean score in those who began receiving in-home care later after discharge, suggesting the need for a range of proposals for establishing care plans when introducing in-home care services. An elderly person who suddenly becomes disabled will have a far more difficult time imagining capably living at home immediately after hospital discharge than one who has experienced a gradual deterioration in ability to function at home while receiving therapy through hospital visits. Individuals discharged from the hospital after becoming disabled must learn to accept their situation as a turning point in their lives and learn to cope with their disability. However, Myerhoff maintains that all elderly individuals have an innate ability to recover continuity in their lifestyle despite facing a crisis, and are capable of maintaining social interactions with important people in their lives, connections to places they have lived, and beliefs in their religion or faith of choice (Myerhoff and Simic 1978). Individuals facing such a critical turning point in their lives therefore need people to support and watch out for them until they recover the ability to carry out functions for daily living. As such, the earnest expertise offered by in-home care staff can be of great service to the elderly in these sorts of situations.

Another factor found to influence QOL of elderly people in the present study was family structure. Structural changes in industry and urbanization over the past 50 years have led to a trend toward nuclear families, and indeed, in our study, single-living and married couple households accounted for 68% of all participants. However, we noted that QOL scores among participants who lived with three generations were significantly high (p = 0.001) compared with living alone, as a couple, or with two generations in the same household. Erikson described the development reciprocity and generation nature as a lively exchange and demonstrated the importance of the involvement of different generations in the developmental issues associated with old age (Erikson et al. 1986). Another factor cited in improving QOL is for a client to discuss his or her views on grandparenthood with therapists of other generations during in-home rehabilitation at home (Miyuki 2009). While altering a patient’s family structure is no easy feat, elderly people living alone or as married couples can still engage in these lively exchanges with in-home care staff members of different generations, using their interactions to learn about each other, grow, and evolve as a result of such care services. These interactions may help improve QOL among elderly people with a family structure other than a three-generation household. Elderly individuals preparing to use in-home care services have concerns and expectations regarding such services.

Our present findings suggest that QOL of elderly people may be improved if in-home care services are provided with consideration for factors known to influence QOL and the consumers’ intentions. We will conduct follow-up research on how QOL of elderly people changes after starting to use in-home care services (Imanishi et al. 2015).

## Conclusions

Here, we reported on the primary effect of starting to use an in-home care service on QOL among elderly individuals. Treatment history (visiting a hospital and receiving therapy but deciding to use in-home care services later) and family structure (three generations) were the two main factors that affected a QOL in our study population. Further, with regard to treatment history, PGC-MC scores were higher in those who visited a hospital and received treatment but decided to use in-home care services later than in those who decided to enroll in the care service while still in the hospital. In contrast, with regard to family structure, PGC-MS scores decreased in the order of three-generation families, those living alone, couples, and two-generation families. Taken together, these findings demonstrate a clear demand for support among seniors that eases the anxiety that accompanies being discharged from the hospital, particularly among seniors with low QOL and in-home care immediately after hospital discharge and those living in a two-generation household. However, while our findings have clarified major causes affecting QOL among elderly individuals, we were unable to identify the root cause of decreased QOL in our population. We hope to address this issue in future research.

## Appendix

Contents of the PGC-MS instrument. Answers are given as yes or no and scored as one point for yes, zero points for no, for a maximum of 17 points. The English depicted below is the original English version of the questionnaire developed by M.P. Lawton.

1. Things keep getting worse as I get older.
2. I have as much pep as I had last year.
3. How much do you feel lonely?
4. Little things bother me more this year.
5. I see enough of my friends and relatives.
6. As you get older, you are less useful.
7. I sometimes worry so much that I can’t sleep.
8. As I get older, things are (better/worse) than I thought they would be.
9. I sometimes feel that life isn’t worth living.
10. I am as happy now as I was when I was younger.
11. I have a lot to be sad about.
12. I am afraid of a lot of things.
13. I get mad more than I used to.
14. Life is hard for me much of the time.
15. How satisfied are you with your life today?
16. I take things hard.
17. I get upset easily.
